# Maritime climate influence on chaparral composition and diversity in the coast range of central California

**DOI:** 10.1002/ece3.1211

**Published:** 2014-09-04

**Authors:** Michael C Vasey, V Thomas Parker, Karen D Holl, Michael E Loik, Seth Hiatt

**Affiliations:** 1 Department of Environmental Studies, University of CaliforniaSanta Cruz, California; 2 Department of Biology, San Francisco State UniversitySan Francisco, California; 3 California State University GIS Specialty Center, San Francisco State UniversitySan Francisco, California

**Keywords:** Local endemism, Mediterranean-type climate, moisture gradient, obligate seeder, summer marine layer

## Abstract

We investigated the hypothesis that maritime climatic factors associated with summer fog and low cloud stratus (summer marine layer) help explain the compositional diversity of chaparral in the coast range of central California. We randomly sampled chaparral species composition in 0.1-hectare plots along a coast-to-interior gradient. For each plot, climatic variables were estimated and soil samples were analyzed. We used Cluster Analysis and Principle Components Analysis to objectively categorize plots into climate zone groups. Climate variables, vegetation composition and various diversity measures were compared across climate zone groups using ANOVA and nonmetric multidimensional scaling. Differences in climatic variables that relate to summer moisture availability and winter freeze events explained the majority of variance in measured conditions and coincided with three chaparral assemblages: maritime (lowland coast where the summer marine layer was strongest), transition (upland coast with mild summer marine layer influence and greater winter precipitation), and interior sites that generally lacked late summer water availability from either source. Species turnover (*β*-diversity) was higher among maritime and transition sites than interior sites. Coastal chaparral differs from interior chaparral in having a higher obligate seeder to facultative seeder (resprouter) ratio and by being dominated by various *Arctostaphylos* species as opposed to the interior dominant, *Adenostoma fasciculatum*. The maritime climate influence along the California central coast is associated with patterns of woody plant composition and *β*-diversity among sites. Summer fog in coastal lowlands and higher winter precipitation in coastal uplands combine to lower late dry season water deficit in coastal chaparral and contribute to longer fire return intervals that are associated with obligate seeders and more local endemism. Soil nutrients are comparatively less important in explaining plant community composition, but heterogeneous azonal soils contribute to local endemism and promote isolated chaparral patches within the dominant forest vegetation along the coast.

## Introduction

California chaparral is a vegetation type with an extensive diversity of woody evergreen shrub species (Cooper 1922; Epling and Lewis 1942; Keeley and Keeley 1988). Other Mediterranean-type climate (MTC) regions also are global biodiversity hot spots with a richer floristic diversity compared with other temperate zone vegetation (e.g., fynbos in South Africa and kwongan in Southwestern Australia) (Myers et al. 2000). Processes influencing this diversity relate in part to long, hot, and dry summers and short, mild, and wet winters characteristic of MTC. Woody plant growth is stimulated by rainfall during the wet season which leads to biomass accumulation, but plants then dry out during the long dry season. As a result, canopy-driven wildfires are an inherent feature of these shrublands (Keeley et al. 2012) and fire adaptations are presumed to have stimulated species diversification in many lineages (Wells 1969; Cowling et al. 1996). Variation in soils has also contributed to plant diversity in all of these regions. Edaphic diversity and plant endemism is substantial in fynbos and kwongan shrubland (Ojeda et al. 2001; Hopper 2009; Keeley et al. 2012) as well as in California chaparral (Wells 1962; Keeley 1992; Anacker and Harrison 2012). In this study, we examine the role of geographical variation in the extent of summer fog and low marine cloud stratus (the summer marine layer) as an additional influence on the compositional diversity of chaparral along the central coast of California.

The summer marine layer in California occurs along the margin of the Pacific coast where it varies daily, yet it creates a persistent, repetitive pattern in dry season water availability each year. Relatively warm, humid winds move onshore from the Pacific Ocean and typically condense into fog and low cloud stratus over deep, cold, and upwelled water adjacent to the coast. A temperature inversion caused by high elevation anticyclonal air masses moving offshore constrains this cool cloud layer to a relatively low elevation (Koraček et al. 2014). The average upper elevation of the summer marine layer is approximately 400 m (Johnstone and Dawson 2010), but it varies depending on local conditions. Also, at different times of the day, it can advect far inland where gaps exist in the steep mountainous terrain that characterizes the California coast. Earlier botanists anecdotally noted the summer marine layer as an important factor influencing coastal vegetation composition and water availability (Shreve 1927; Hoover 1970; Howell 1970; Griffin 1978), but its ecological significance was generally not well appreciated (e.g., Richerson and Lum 1980). However, recent studies have demonstrated that significant enhancement of dry season plant water availability for coastal vegetation can occur in a variety of ways, including water supplements from fog drip (Dawson 1998), foliar uptake (Burgess and Dawson 2004; Limm et al. 2009), reduced evapotranspiration (Williams et al. 2008; Fischer et al. 2009) and enhanced microbial activity in fog-influenced soils (Carbone et al. 2012).

Species diversity and local endemism appear to correlate with climate gradients in other MTC regions such as South Africa (Cowling et al. 2005) and southwestern Australia (Lamont et al. 2002). The highest levels of local endemism in diverse shrubby genera such as *Erica* (Ojeda 1998; Ojeda et al. 2005) and *Banksia* (Lamont and Connell 1996) are associated with more favorable soil water availability, whereas more severe, drought-prone areas are characterized by fewer and more widespread species. California has the most extreme dry season of all MTC regions (Cowling et al. 2005) where, on average, only five percent of its rainfall occurs from May through September. Patterns of diversity in California vegetation also can vary with differences in climate characteristics, for example, through timing of rainfall events, precipitation amount, and subsequent soil water availability (Loik et al. 2004), but regionally scaled diversity patterns of chaparral have previously not been assessed in this regard.

**Figure 1:**
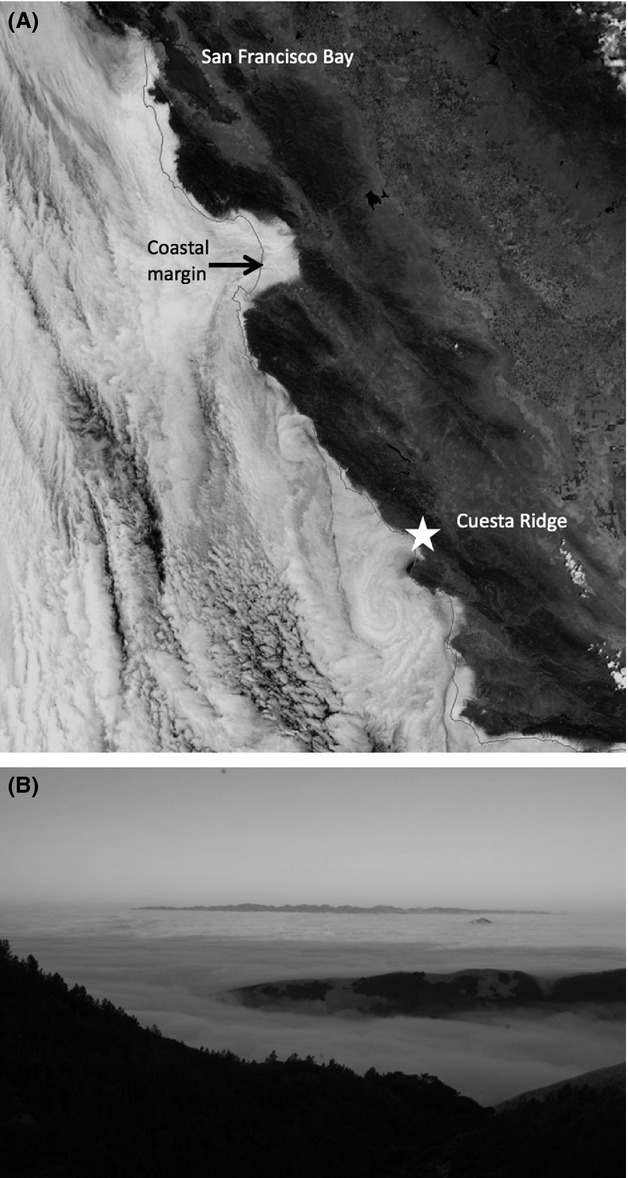
Geographical relationship of the summer marine layer to two key variables: *distance from the coast* and *elevation*. (A) Satellite view of Central West Region of California during a typical summer day. Low elevation summer marine layer abuts coastal uplands and advects inland through gaps in the coast range. Cool, moist air flows inland daily and then back coastward over tens of kilometers across the landscape. (B) View southwest from Cuesta Ridge sample site over the city of San Luis Obispo [illustrated in (A), ~ 720 m elevation] toward the Irish Hills and the Pacific Coast, San Luis Obispo County (Photo by M.C. Vasey). Low elevation marine layer is trapped below a warm and dry inversion air mass which typically moves several meters up and down on a daily basis. Cuesta Ridge, an upland coastal chaparral site, hosts a fire-dependent, local endemic evergreen shrub, *Arctostaphylos luciana*. Beneath the fog layer, nearby lowland coastal chaparral sites host other local endemics such as *A. pechoensis*, *A. morroensis*, *A. osoensis*, and *A. cruzensis* (see Table S2 for species authorities).

**Figure 2:**
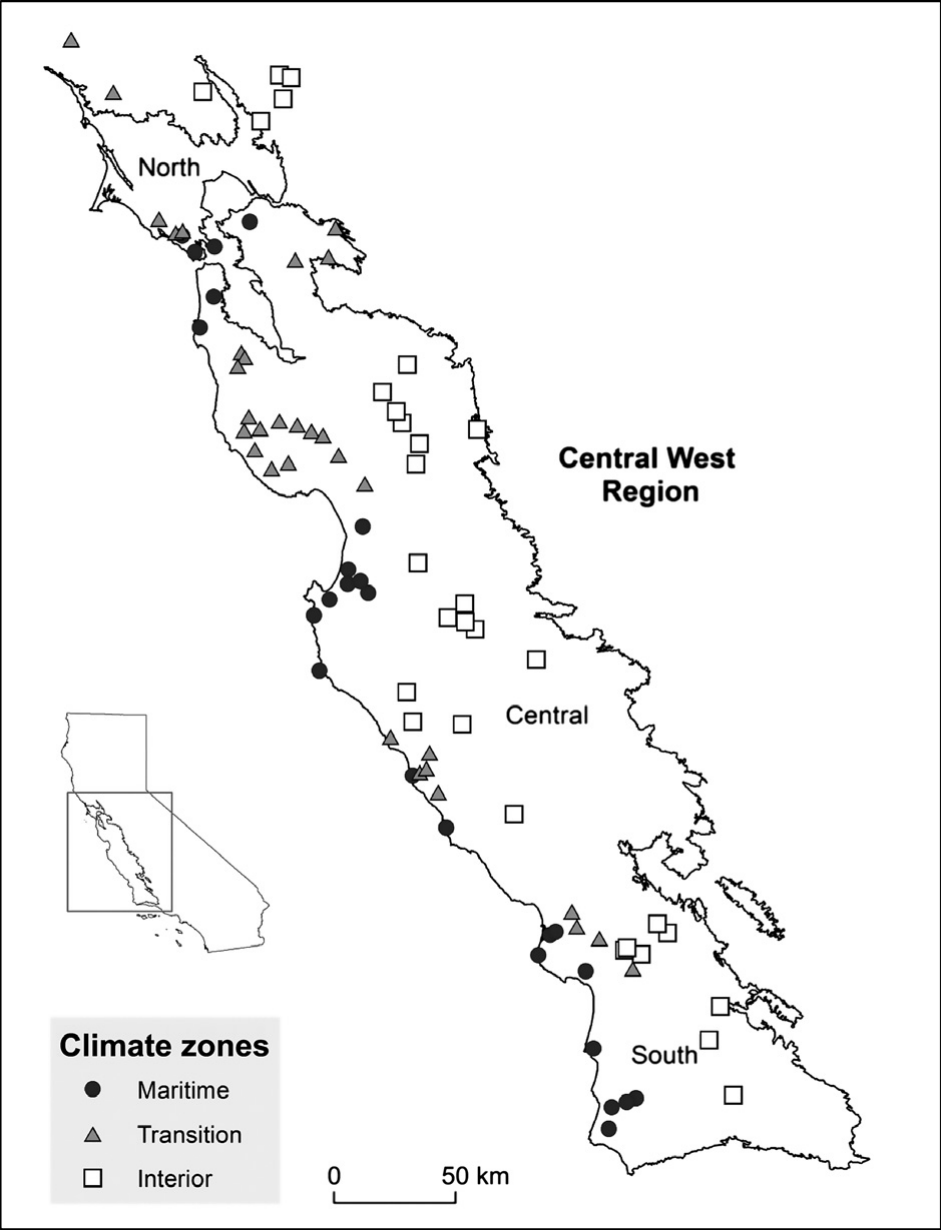
Sample sites (87) in the Central West Region of California. Based on cluster analysis of ten climate variables (emphasizing summer dry season variables), the sites clustered into three climate zones: maritime (*n* = 25), transition (*n* = 32), and interior (*n* = 30). Projection is California Teale Alber’s (NAD 83).

Chaparral is one of California’s most widespread vegetation types and, unlike fynbos or kwongan shrublands, most species in California chaparral are widespread while local endemics are relatively uncommon (Keeley and Davis 2007). A major exception to this pattern is the central coast mountain ranges, especially a narrow zone of chaparral that occurs in lowlands and uplands situated within a short distance of the Pacific Ocean (Fig. 1A and B) (Cody 1986; Keeley 1992). *Arctostaphylos,* for example*,* is the most diverse shrub genus in California chaparral (Parker et al. 2012) and contains numerous local endemics along the central California coast (Vasey and Parker 2008). The lowland portion of this vegetation has been called “maritime chaparral” (Griffin 1978), harbors numerous local endemic woody species, and consequently is of special conservation concern (Sawyer et al. 2009). Although maritime chaparral is distributed from approximately Mendocino County to northern Baja California, Mexico (Sawyer et al. 2009), the most extensive expression of maritime chaparral diversity occurs in central California, ranging from the counties of Sonoma (38°N latitude) to Santa Barbara (34°N latitude), an area referred to as the Central West Region (Davis et al. 1998; Fig. 2). Because of the summer marine layer, substantial climate differences exist during the dry season over short distances from coast to interior; that is, the immediate coast is relatively cool and moist in summer, while conditions get hotter and dryer with increases in elevation and distance toward the interior (Abatzoglou et al. 2009; Johnstone and Dawson 2010). A water availability gradient associated with the summer marine layer is consistently present each year with, for example, significant differences in end-of-dry season water potentials for *Arctostaphylos* species from coast-to-interior sites (Vasey et al. 2012).

In this study, we hypothesize that chaparral compositional diversity and *β*-diversity (species turnover as an indicator of local endemism [Whittaker 1960; Harrison et al. 2011]) will correspond with more favorable water availability conditions associated with the summer marine layer in the Central West Region of California. We also examine other climatic factors known to influence coastal vegetation, such as total annual rainfall (Richerson and Lum 1980) and minimum temperatures during the coldest month associated with winter freeze events (Ewers et al. 2003). We further test whether soils and/or topography are alternative factors more strongly associated with these compositional diversity patterns. Finally, we relate our findings to the larger context of fire regimes and edaphic diversity and explore implications for conservation.

## Methods

### Vegetation composition

We sampled vegetation at 87 sites in the Central West Region of California (Davis et al. 1998) from March to September in 2008 and 2009 (Fig. 2, Table S1). Sampling was restricted to recognizable perennial species (ferns, geophytes, perennial herbs, vines, subshrubs, shrubs, and trees). Ephemeral annuals were excluded because they are uncommon in mature chaparral and not visible during portions of the time span when water availability is most limiting and when most sampling occurred. Species were identified as minimum taxonomic units, including subspecies and varieties and are hereafter referred to as “species” for the sake of simplicity. Where species could not be identified in the field, we made field collections and determined their identity based on local floras and the Jepson Manual (Baldwin et al. 2012). A total of 238 species were identified in these samples (Table S2).

Sites were located based on accessibility and with the objective of distributing sites throughout the region. When it was possible to identify sites in advance, they were divided into 1 km^2^ grids on Google Earth, and the grids were selected using a random number table. When sample sites were identified in the field, random access points were identified. A random number table was used to count out a number of paces to establish a plot origin. Origins were then established at least 5 m off the edge of the trail, fire road, or road. Because the scale of inquiry was regional in scope and the goal was to represent each site equally (Whittaker et al. 2001), only one 0.1 ha plot was sampled per site throughout the region. We utilized a modified Keeley plot (Keeley and Fotheringham 2005) for our sampling design consisting of a 0.1-ha rectangular plot (20 × 50 m) divided into ten 10 × 10 m (100 m^2^) subplots. Given the minimal occurrence of herbaceous species under mature chaparral canopies, we did not sample 1 m^2^ plots. Within each 100 m^2^ subplot, we estimated cover of individual species using a modified Daubenmire (1959) cover class system (0–1%, 1–5, 5–15, 15–25, 25–50, 50–75, 75–95, and 95–100). For each plot, we recorded spatial coordinates at the origin and measured elevation (m), slope, and aspect.

### Soil

A 10 cm deep and ~30 g soil sample was collected at the center of each subplot after removing the litter layer. The subplot samples were pooled, air-dried, lightly crushed with a pestle, and well mixed. Samples were passed through a 2.0-mm sieve and analyzed by Brookside Labs (New Knoxville, OH, USA; www.blinc.com/worksheet_pdf/SoilMethodologies.pdf). Sulfur, Na, K, Ca, Mg, Fe, and Al were extracted using a Mehlich III extractant. Phosphorus was extracted using the Bray method. Percent organic matter was calculated based on loss upon ignition, and N levels were derived from % organic matter. Soil analysis also included pH (1:1 in H_2_O) and total cation exchange capacity. Soil texture was determined using the method of Kettler et al. (2001).

### Climate

Climate variables were estimated for each plot based on four data sources referenced to each plot’s spatial coordinates. Summer cloud frequency for each plot was obtained from Williams (2006), who used daily MODIS imagery collected by the Terra satellite to calculate spatially continuous (250 m resolution) fields of summertime mid-morning (10:30 PST) cloud frequency between Julian days 184 and 274 (i.e., July 3–October 1) for the California coast during 2000–2006. Mean cloud-frequency values were extracted for each plot from coincident (or nearest) raster cells for all 7 years. The maximum cloud frequency pixel value from these 7 years was assigned to approximate the greatest influence of the summer marine layer for each plot.

Additional climate data were downloaded from the PRISM website (www.prism.oregonstate.edu) which provides 30-year monthly averages from 1971 to 2000 using 800-m grid cells. At each plot location, average monthly precipitation, average maximum temperature, average minimum temperature, and average dew point were extracted from PRISM grid cells. Average annual precipitation (*Precip*) was calculated by adding average monthly precipitation for each water year (October–September). We calculated potential evapotranspiration (*PET*) based on PRISM data. A series of ARC Macro Language (aml) files were used to produce climate grids that served as inputs to a water balance calculation (Jensen and Haise 1963) for each plot. The original inputs included precipitation, temperature, and solar radiation. Those variables were then used to generate grids of PET in cm month^−2^. We also downloaded climate variables for the spatial coordinates of each plot from WorldClim based on data from 1950 to 2000 at a 1-km resolution (www.worldclim.org/bioclim). These variables include temperature seasonality, mean maximum temperature of the warmest month, mean minimum temperature of the coldest month, and precipitation seasonality.

Temperature and RH are the two climate variables that drive atmospheric evaporative demand, as reflected in *VPD* and *Ψ*_atm_ (Nobel 1991). To estimate *VPD* and *Ψ*_atm_ for each of our plots, we used CIMIS (www.cimis.water.ca.gov/cimis) and RAWS (www.raws.dri.edu) dry season (May–September) temperature and RH data from 93 meteorological stations in the central California coast region that include our sample plots. These data were gathered over 13.2 ± 5.9 (mean ± SE) years. We calculated *VPD* and *Ψ*_atm_ for each of the meteorological stations based upon Nobel (1991). We developed a multiple regression model using elevation and distance from the coast for each station as independent variables against *VPD* and *Ψ*_atm_ separately as dependent variables. Distance of the plot from the coast was calculated based on Euclidean distance. The models were both significant (*Ψ*_atm_ *r*^2^ = 0.68, *P* < 0.0001; *VPD r*^2^ = 0.65, *P* < 0.0001). Using the slope and intercept from each regression, we estimated *VPD* and *Ψ*_atm_ for each plot based on their elevation and estimated distance from the coast based on the equations: *VPD* = 0.4913554 + (elevation*0.0006084) +(distance*0.010729) and *Ψ*_atm_ = −1*(37.138109 + (elevation*0.0602212) + (distance*0.6098197). For the four plots for which we had micrometeorological data over three successive dry seasons (Vasey et al. 2012), we calculated *VPD* and *Ψ*_atm_ directly.

### Data analysis

Thirty-one environmental variables (Table S3) were analyzed using principal components analysis (PCA). Where appropriate, these variables were transformed using log, square root, and arcsine square root functions to meet assumptions of homoscedasticity. Data were normalized by subtracting the mean from each variable and dividing by the standard deviation. We compared all environmental variables by climate zones using a one-way ANOVA.

As our focus was on regional climate differences among plots, we then ran a cluster analysis on plots using 10 climate variables identified by the PCA as having high eigenvalue loadings (Table S3); variables included average daily dry season *Ψ*_atm_, *VPD*, *PET*, *T*_max_ for the warmest 3 months of the year (June–August), *T*_min_ for the three coldest months of the year (December–February), percent dry season cloud frequency (*CF*), mean maximum temperature during the hottest month of the year (*MTW*), mean minimum temperature during the coldest month of the year (*MTC*), average annual precipitation (*Precip*) and temperature seasonality (*TS*). Data were normalized and a matrix of Euclidean distances between each pair of variables was calculated. We conducted a group average cluster analysis on this distance matrix and identified three climate zone groups that emerged from the plots (See Fig. S1).

Nonparametric procedures were used to conduct multivariate analyses on the vegetation cover matrix because the matrix was zero rich and not normally distributed. We square-root-transformed cover data to give more weight to species with low cover value and computed a Bray–Curtis percent dissimilarity measure for each plot. To assess the relationship of vegetation composition data to climate zones determined by the cluster analysis of environmental variables, we ran a multiresponse permutation procedure (MRPP) using the square–root-transformed Bray–Curtis distance matrix. We calculated richness, evenness, Shannon Diversity Index (*H’*), and Simpson Diversity Index (*D’*) for each plot and compared these across climate zones using a one-way ANOVA.

A nonmetric multidimensional scaling (NMDS) analysis was performed on the transformed species matrix. We selected three dimensions with 500 runs including randomization tests plotting stress versus iteration with varimax rotation to orthogonalize the three axes. Five separate NMDS procedures were performed with a random number of seeds plus 500 runs of real data. All produced similar results including similar stress levels (~16.8). To calculate *β*-diversity (species turnover) within zones, we transformed the raw cover data matrix using log_10_ (*x*) + 1 and then conducted a multivariate analysis of dispersion in R using the “betadisper” script in the Vegan package (Anderson et al. 2006). The most important vector of environmental variables per plot (PC1) was then regressed against the strongest plot composition dissimilarity vector (NMDS 2) to examine the relationship between these variables and composition dissimilarity between plots.

We used the Central West Region results from the California Gap Analysis by Davis et al. (1998; Appendix CW, Table CW-2) to estimate the amount of area covered by chaparral in the three climate zones identified in the cluster analysis. Twenty-four chaparral and related closed-cone conifer natural communities (Holland 1986) were assigned to these zones based on canopy dominants. The total areal distribution (km^2^) of each natural community was then summed for each zone.

All species were categorized by life form: trees, shrubs, subshrubs, perennial forbs, perennial graminoids, ferns, or vines, and we calculated the proportion of total cover for each life form in each climate zone. Shrubs were also grouped in two ways: first, by the three postfire recruitment-dependent genera (*Arctostaphylos, Ceanothus,* and *Adenostoma*) while all other shrub species were grouped as “Other”. Second, shrub species were grouped by postfire life history modes into obligate seeders, facultative seeders (resprouters), and obligate resprouters (Keeley et al. 2012). Cover values for shrub genera were square-root-transformed. Cover of shrub genera and shrub postfire life history modes were compared by climate zone groups using a one-way ANOVA and *post hoc* Tukey HSD tests. Numbers of species recorded in sample plots for each climate zone were calculated including unique species, species shared between different zones, and species shared by all three zones. Numbers of special status species, a surrogate for species with a restricted distribution, were calculated based on the California Natural Diversity Data Base (www.dfg.ca.gov/biogeodata/cnddb).

## Results

**Figure 3:**
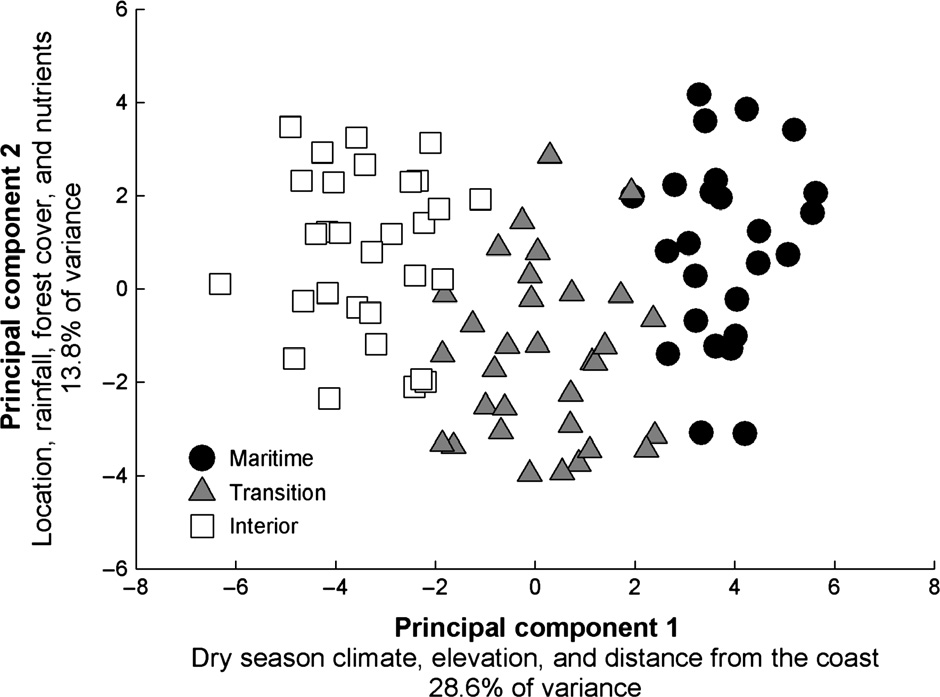
Principal Components Analysis based on 31 environmental variables obtained from each plot (Table S3). Plots are identified by climate zone groups derived from cluster analysis (Fig. S1).

In the PCA of environmental variables, sample plots were well defined by climate zone groups along the first axis (28.6% of variance explained, Fig. 3) with six climate variables associated with the summer marine layer generally having the highest eigenvalues. Climate zone groups [labeled “maritime,” “transition,” and “interior,” consistent with the geographical location of plots from coastal lowlands, coastal uplands, and interior uplands, respectively (Fig. S1)], were derived by Cluster Analysis utilizing climate variables with the highest loading values identified in the PCA. In addition to climate factors associated with summer temperature, relative humidity, and cloud frequency in PC1, elevation and distance from the coast also had high loading values along the first axis. Temperature seasonality and mean minimum of the coldest month of the year had high loading values along this axis as well (Table S3). The second PCA axis (13.8% of the variation) was most strongly associated with latitude, longitude, mean annual precipitation, and soil nutrients such as P, S, Na, and Ca. The third PCA axis (9.2% of the variance) related to soil texture and pH.

**Figure 4:**
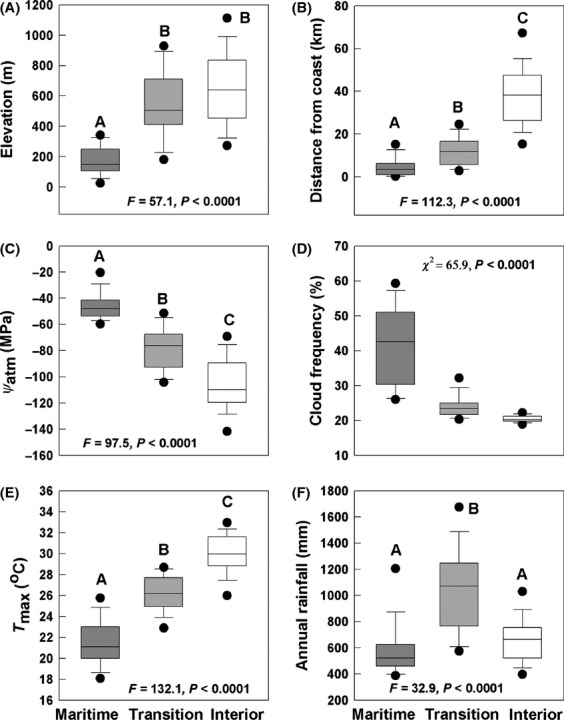
Environmental variables grouped in different dry season climate zones. (A) elevation; (B) distance from the coast; (C) mean daily dry season atmospheric water potential (*Ψ*_atm_); (D) percent of time cloud cover is present at sample plots at midmorning during the summer dry season; (E) average daily maximum temperature during June–August over a 30-year interval; (F) average total annual rainfall over a 30-year interval interpolated for each plot. Capital letters represent significant differences between zones based on Tukey’s HSD *post hoc* tests. Box plot represents median, 25th–75th percentile (box outline), 5th–95th percentile (whiskers), and maximum and minimum values beyond the 5th –95th percentiles (circles).

Maritime sites had more summer cloud cover, were cooler in the summer, and had less dry season evaporative demand than inland sites (Fig. 4A–E). Transition sites were intermediate in variables related to dry season evaporative demand. However, annual rainfall was significantly greater in the transition zone than the maritime and interior zones (Fig. 4F). Both transition and interior zone sites were higher in elevation than maritime sites (Fig. 4A). As expected, *Ψ*_atm_, cloud frequency, and *T*_max_ were strongly correlated with other dry season evaporative demand variables, such as *VPD*, *TS*, *PET*, and *MTW* (*r* ≥ 0.65 for all pairwise correlation coefficients, *P* < 0.0001) as well as with *MTC*.

**Figure 5:**
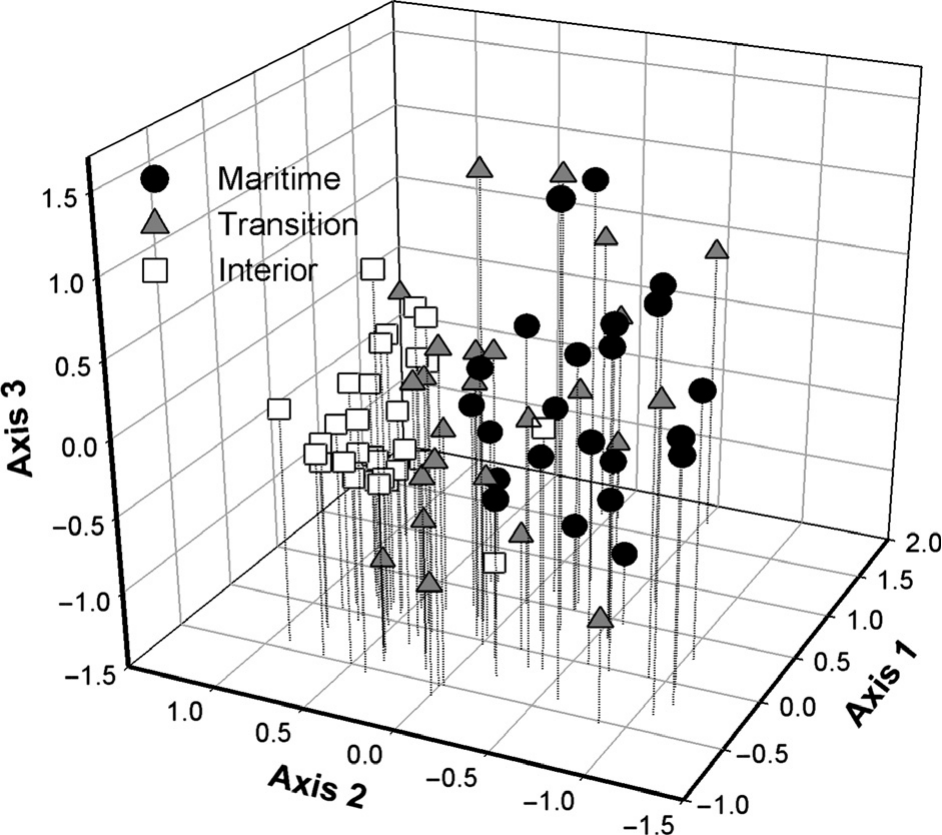
Nonmetric multidimensional scaling analysis results of vegetation community composition with plots identified by climate zones. Plots are grouped on the basis of pairwise Bray–Curtis dissimilarities of a square root-transformed species (238) by plot (87) matrix.

The NMDS analysis of vegetation composition data illustrated that chaparral assemblages vary across the three climate zones (Fig. 5). The interior plots were clustered closely together, whereas transition and maritime plots were more scattered, indicating that there are greater compositional differences among plots within transition and maritime zones compared to the interior zone. The MRPP analysis for vegetation strongly supported the difference between climate zones (*T* = −18.8, *P* < 0.0001, *A* = 0.05). All pairwise comparisons between climate zones were significant with maritime and interior sites being most different (*T* = −19.1, *P* < 0.0001, *A* = 0.07), and maritime and transition locations the least different (*T* = −5.8, *P* < 0.0001, *A* = 0.02). The multivariate analysis of dispersion (*F* = 6.7, *P* = 0.002) and pairwise Tukey’s HSD *post hoc* tests showed that maritime and transition plots both had significantly higher dispersion in community composition among plots (*β*-diversity) compared with the interior zone, but did not differ significantly from one another. Environmental variables associated with the summer marine layer (PC1) were found to be strongly correlated with the strongest among plot composition dissimilarity vector (NMDS 2) (*r*^2^ = 0.55, *P* = 0.0001).

**Figure 6:**
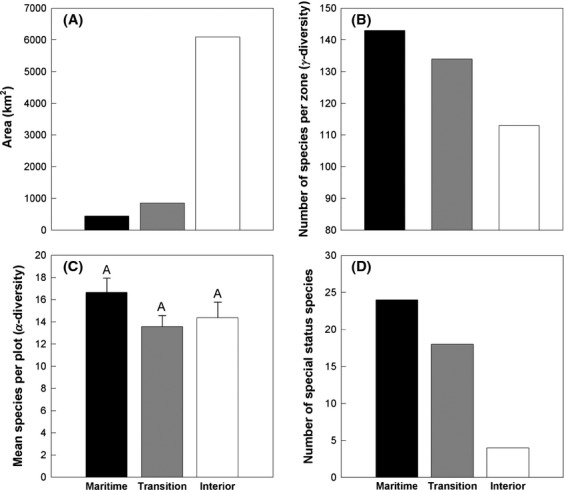
(A) Estimate of climate zone area in Central West Region covered by chaparral; (B) total number of species per zone (*γ*-diversity); (C) mean number of species per plot (± SE) (*α*-diversity); and (D) number of special status species per zone. Means with the same letter are not significantly different (*P* < 0.05) using Tukey’s HSD test.

Of the total area of the Central West Region covered in chaparral, approximately 82% occurs in the interior zone, 12% in the transition zone, and only 6% in the maritime zone (Fig. 6A). Yet, the total number of species (*γ*-diversity) recorded in the maritime (143) and transition zones (134) were higher than the interior zone (113) (Fig. 6B). The mean within-plot species richness (*α*-diversity; Fig. 6C), species evenness, *H’*, and *D’* were not significantly different among climate zones. The number of special status species in the maritime zone (24) and transition zone (18) was substantially greater than the interior zone (4) (Fig. 6D). Species unique to a single climate zone were mostly found in the maritime zone with nearly twice the number compared with other climate zones (Table S2). Fifty species (21%) occurred in all three zones.

**Figure 7:**
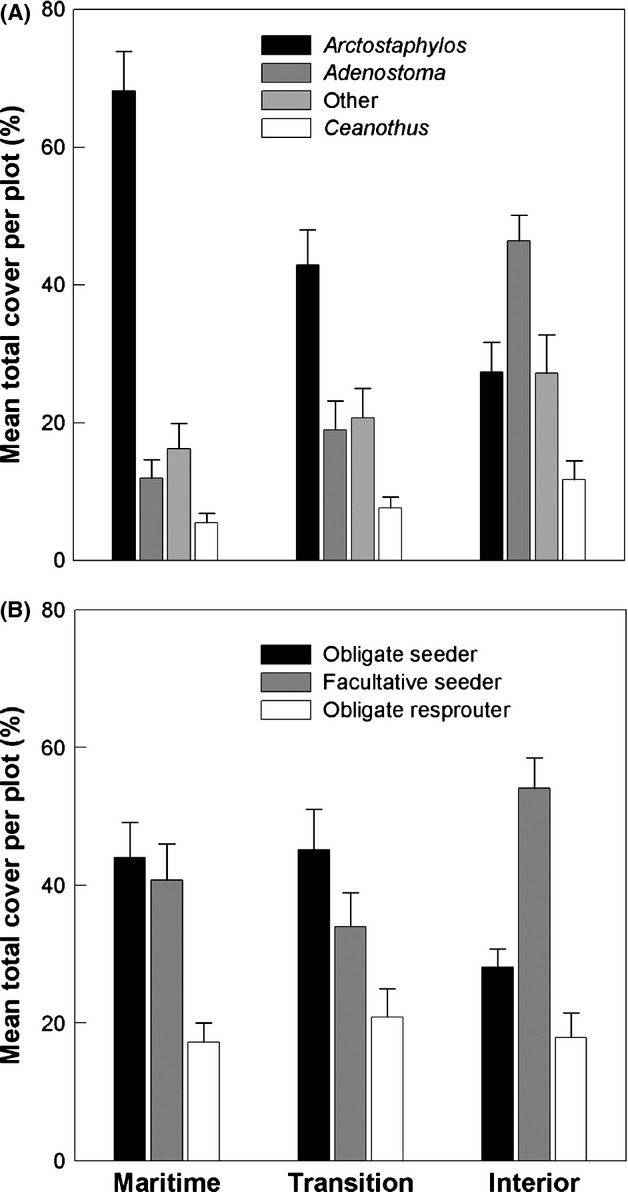
Mean percent of total cover per plot (± SE) of different shrub species in the three climate zones categorized by (A) the most abundant genera and (B) three different postfire life history modes. Means with the same letter are not significantly different (*P* < 0.05) using a Tukey’s HSD test.

Shrubs constituted 78% of the vegetation cover across all plots and, of the 89 shrub species recorded, 79% of the shrub cover was comprised of species in three genera (*Adenostoma, Arctostaphylos,* and *Ceanothus*) that rely upon postfire conditions for seedling recruitment. Most of the remaining 21% shrub cover was comprised of obligate resprouters (although 2% were obligate seeder species from genera other than the three listed above). *Arctostaphylos* species dominated cover in the maritime zone, whereas *Adenostoma* dominated in the interior zone (Fig. 7A). Obligate seeder shrub cover was greatest in the maritime and transition zones, whereas resprouting facultative seeders dominated cover in the interior zone and obligate resprouter cover was similar in all zones (Fig. 7B).

## Discussion

Chaparral in the central coast region of California tends to shift from *Arctostaphylos* dominated chaparral in coastal sites to *Adenostoma* dominated chaparral in the interior. The coastal lowlands and uplands further harbor considerable endemism, especially within *Arctostaphylos* and to a lesser extent in *Ceanothus*, and as a consequence display greater *β*-diversity than chaparral in the interior. Although the average number of species per plot was not significantly different across the three climate zone groups, coastal lowlands and uplands contained greater *γ*- and *β*-diversity and over 90% of the special status chaparral species for the central coast (Figs. 5, 6). At the same time, the coastal chaparral assemblages comprise only about 18% of total spatial distribution of chaparral in the Central West Region coastal ranges (Fig. 6A), suggesting greater species turnover and local endemism of coastal chaparral per unit area than interior chaparral. This compositional and *β*-diversity gradient is responding to a complex set of climatic factors influenced by proximity to the Pacific Ocean.

A pronounced water availability gradient characterizes the central coast of California during the rainless summer months (Vasey et al. 2012; Figs. 3, 4) that generally corresponds with this pattern of woody plant composition and *β*-diversity. In coastal lowlands, the cool and moist conditions of the summer marine layer reduce evaporative demand (atmospheric drought as shown by *PET* and *Ψ*_atm_) and likely supplement moisture availability (via fog drip or foliar uptake) that offsets water deficits due to low annual rainfall. In coastal uplands, the summer marine layer influence on atmospheric drought is weaker, but coastal uplands receive greater annual rainfall than either coastal lowlands or interior uplands. Interior uplands are minimally affected by the summer marine layer influence and receive much less annual rainfall than coastal uplands (Fig. 4).

This summer water availability gradient is complex because it involves both soil drought due to minimal annual rainfall (coastal lowlands and interior) and atmospheric drought due to hot and dry summer conditions (interior and coastal uplands to some extent). At the end of the dry season, vegetation from interior locations experiences the combined impact of both of these ecohydrologic conditions (Bhaskar and Ackerly 2006), which is possibly why fewer woody evergreen species are able to survive in the coastal interior. Consequently, rather than a gradual transition of chaparral species composition from coast to interior, there appears to be a significant break between more mesic coastal chaparral (lowland and upland) on the one hand and more xeric interior chaparral on the other.

The maritime influence on regional temperature extremes in the coast region is also well recognized (Abatzoglou et al. 2009). Winter freeze events may also be a factor in coastal chaparral distribution (Ewers et al. 2003), as suggested by the high loading value of the minimum temperature of the coldest month of the year in PC1 (Table S3). Winter freeze events and extreme summer drought probably combine to constrain chaparral diversity in the interior where species tend to be fewer and more widespread (Fig. 6). Thus, coast range chaparral vegetation patterns appear to correlate with the summer marine layer, enhanced summer water availability, increased annual rainfall, and vulnerability to winter freeze events; that is, the relative importance of these different climatic factors are probably all intertwined and may differentially impact particular species in different local venues.

Drivers of chaparral endemism in this region are also doubtless complex (Stebbins and Major 1965; Raven and Axelrod 1978; Anacker et al. 2011). Indirectly, the reduction in summer water deficit by the marine layer permits more mesic vegetation such as coastal forests to dominate many coastal landscapes, contributing to the heterogeneity of coastal chaparral by isolating chaparral stands in archipelagoes of unproductive topographic and edaphic settings (Griffin 1978; Kruckeberg 2002). This water availability gradient also generates a gradient of live fuel moisture content in chaparral stands (Vasey, unpublished) and therefore contributes to longer fire return intervals in the coastal zone (Odion and Tyler 2002; Dennison et al. 2007). Although conifers and oaks may invade coastal chaparral (Horton et al. 1999; Van Dyke et al. 2001), the two vegetation types tend to coexist due to different fire regimes. When fires occur, the divergence in fire intensity between chaparral and forest reinforces the presence of chaparral by excluding forest trees (Odion et al. 2010).

Chaparral is a fire-adapted vegetation type and all three climate zones are characterized by the dominance of species with persistent soil seed banks (~ 80%) that depend upon fire to stimulate germination. Fire regimes vary among these different zones, however, and a legacy of increased water availability interacting with longer fire return intervals may be the driver of increased prominence of obligate seeder species in coastal chaparral stands. Postfire obligate seeder (OS) shrubs are hypothesized to be favored in habitats with moderately long fire return intervals and intense burns (Keeley and Zedler 1978) and, with some exceptions, this hypothesis has been supported in various MTC shrubland habitats (reviewed by Bell 2001). Obligate seeders represent about two-thirds of the species in the California chaparral genera *Arctostaphylos* and *Ceanothus*, which are the most species-rich genera in chaparral (Wells 1969; Baldwin et al. 2012). A large majority of obligate seeding species are local endemics, and they drive much of the observed *β*-diversity patterns. Coastal chaparral plots tend to be codominated by obligate seeder and facultative seeder shrubs (OS:FS = 1.08–1.10), whereas resprouting facultative seeders dominated interior chaparral (OS:FS = 0.58) (Fig. 7B). This finding is consistent with patterns found along water stress/fire frequency gradients in other MTC shrublands (Ojeda 1998; Bell 2001; Clarke and Dorji 2008).

Central coast chaparral occurs on a wide variety of substrates, yet soil nutrients and texture explained less of the environmental variance at the scale of this study (Fig. 3). Edaphic islands in coastal lowlands and uplands are highly variable in character, ranging across diverse substrates such as deep sands and shallow, rocky outcrops of granite, serpentine, sandstone, conglomerate, and shale. Soil nutrients and texture may be important to coastal chaparral diversity because they create low-productivity habitats favoring the establishment of “islands” of chaparral in maritime landscapes (Kruckeberg 2002) otherwise dominated by forest, grassland, and semi-deciduous coastal scrub (Callaway and Davis 1993). Rather than specific soil types strongly driving chaparral community composition, much of the coastal patterns of diversity may depend on overall edaphic heterogeneity and spatial isolation among relatively small and isolated chaparral stands. Modeling studies examining these types of spatially heterogeneous environments have found that structural heterogeneity is an important driver of speciation via selection regimes that arise both from diversification and refugial processes (Haller et al. 2013).

In summary, central coast chaparral illustrates the complex coupling of near coast influences such as sea surface temperatures and regional vegetation composition and diversity of the adjacent mainland. The maritime influence is complex and in central California involves dimensions of mild temperatures associated with the summer marine layer, enhanced summer water availability, increased annual rainfall on coastal uplands, and reduced vulnerability to winter freeze events near the coast. Dominant genera shift from coast to interior with the greatest diversity and endemism situated in coastal lowlands and uplands. These shifts in species diversity and local endemism parallel patterns in other MTC regions such as South Africa (Cowling et al. 2005) and southwestern Australia (Lamont et al. 2002). The summer marine layer most likely has been an important ecological factor along the California coast for the past few million years (Jacobs et al. 2004; Millar 2012). This has provided ample time to influence evolution within woody shrub lineages, particularly the more rapidly evolving obligate seeder lineages (Wells 1969). Although typically narrow in width, California’s coastal zone extends over 1100 km in length (Abatzoglou et al. 2009) and, consequently, has provided adequate geography for the evolution of local endemics as well as for the establishment of refugial coastal chaparral habitats that favor paleoendemics (Stebbins and Major 1965). As in other MTC regions, this complex of interrelated geologic, climatic, and fire regime factors (Keeley et al. 2012) poses great challenges for the conservation of this diversity, particularly in light of rapid global change.

### Conservation implications

Many maritime chaparral stands are embedded within or adjacent to the rapidly urbanizing coastal region of California. Consequently, loss of habitat through development is a critical near term threat to this endemic-rich ecosystem. For example, serpentine habitat for *Arctostaphylos montana* subsp. *ravenii* and *A. franciscana* in San Francisco practically has been eliminated, and these two taxa now consist of one individual genotype each in “wild” protected and nonchaparral habitat (Parker et al. 2007). Another near term threat is management of the wildland–urban interface to protect human structures from wildfire (Keeley et al. 2012). Fuel breaks and fuel reduction treatments, including mastication and herbicide treatments, collectively can remove many mature shrubs and disrupt the seed bank, disturb associated wildlife populations, and cause other ecological damage to chaparral stands. These near term threats are in part mitigated by federal and state endangered species protection for a few endemics and also by protection of the maritime chaparral community, which is recognized as a special status community under the California Environmental Quality Act and as an “Environmentally Sensitive Habitat Area” under the California Coastal Act.

In this study, we found that lowland coastal chaparral and upland coastal chaparral each contain high numbers of local endemics and correspondingly high *β*-diversity. Considering both lowland and upland coastal chaparral as variants of “maritime chaparral” (Sawyer et al. 2009) seems appropriate because of the coastal influence on both vegetation types. Given that maritime chaparral is recognized as a special status plant community with regulatory protection, this would provide these chaparral areas with regulatory attention during permitting processes.

While habitat preservation and species protection will help in the short term, promoting the combination of processes that likely have sustained diversity in this ecosystem will be difficult. Although increases in human density along the coast will increase fire ignitions (Keeley et al. 1999), most of these fires are controlled and fire risk is actively managed to reduce risk to human structures. This will lead to altered fire return intervals, as well as alternative disturbance regimes (e.g., wet season controlled burns) that will strongly affect chaparral community composition. Climate change will influence fire regimes, but also the processes that mitigate summer water availability on which the vegetation appears to depend (Vasey et al. 2012). Model predictions for coastal regions are of two extremes: The coast may lose fog, advancing the trend already observed over the last century (Johnstone and Dawson 2010), or increase in fog days as the interior heats up (Snyder et al. 2003). Clearly, conservation of maritime chaparral diversity is a multifaceted and multiscaled problem (Whittaker et al. 2005) that invites creative approaches, such as the establishment of conservation refugia in landscaped settings outside of wildlands, and, potentially, assisted migration of lineages to suitable habitats as they materialize over time and as circumstances warrant.

## Supporting Information

**Table S1.** Table of sample plot locations, elevation, and distance to ocean. **Table S2.** Table of species composition (frequency and average cover) for plots by climate zone. **Table S3.** Table of environmental variables sorted by principle component scores. **Figure S1.** Cluster Analysis figure.
