# Supplementary material for: Maritime climate influence on chaparral composition and diversity in the coast range of central California
Source: Ecol Evol. 2014 Sep 4;4(18):3662–74. doi: 10.1002/ece3.1211 (PMC4224539; doi:10.1002/ece3.1211)
Supplement: Supplementary file 1 — Table S1. Table of sample plot locations, elevation, and distance to ocean. Table S2. Table of species composition (frequency and average cover) for plots by climate zone. Table S3. Table of environmental variables sorted by principle component scores. Figure S1. Cluster Analysis figure. [file ece30004-3662-sd1.docx]

**Cover photo caption** Patch of upland coastal chaparral on Butano sandstone outcrop in mid-July nested within a conifer forest matrix along China Grade, Santa Cruz Co. (~ 580 m). Note the interaction of vegetation with low cloud cover. *Arctostaphylos andersonii*, a Santa Cruz mountain endemic, is blooming in the foreground. Two other regional *Arctostaphylos* taxa co-occur at the same site.

**SUPPORTING INFORMATION**

**Table S1** Table of sample plot locations, elevation, and distance to ocean.

**Table S2** Table of species composition (frequency and average cover) for plots by climate zone.

**Table S3** Table of environmental variables sorted by principle component scores.

**Fig. S1** Cluster Analysis figure.

**Table S1** Sample plot information sorted by latitude. M = maritime, T = transition, I = interior, Lat = latitude, Long = longitude, Elev = elevation (m), Dist = distance (km)

| Zone | Site | County | Lat | Long | Elev | Dist |
| --- | --- | --- | --- | --- | --- | --- |
| T | The Cedars | Sonoma | 38.624 | -123.136 | 435 | 15.4 |
| I | Quail Ridge Reserve | Napa | 38.514 | -122.146 | 349 | 42.9 |
| I | Stebbins Reserve | Yolo | 38.505 | -122.088 | 412 | 41.3 |
| I | Sugarloaf SP | Sonoma | 38.445 | -122.505 | 543 | 33.2 |
| T | Harrison Grade | Sonoma | 38.433 | -122.929 | 220 | 14.5 |
| I | Mix Canyon Rd | Yolo | 38.426 | -122.125 | 798 | 32.9 |
| I | Mt George | Napa | 38.341 | -122.228 | 357 | 25.3 |
| T | Bolinas Ridge | Marin | 37.967 | -122.696 | 412 | 3.6 |
| M | Sobrante Ridge | Contra Costa | 37.966 | -122.268 | 208 | 5.8 |
| T | Black Diamond Mines | Contra Costa | 37.951 | -121.864 | 346 | 10.3 |
| T | East Mt Tam | Marin | 37.926 | -122.583 | 665 | 5.0 |
| T | Simmons Trail | Marin | 37.918 | -122.615 | 668 | 3.1 |
| M | Camp Eastwood | Marin | 37.909 | -122.584 | 263 | 4.0 |
| M | Angel Island | Marin | 37.870 | -122.430 | 102 | 0.2 |
| M | Marin Headlands | Marin | 37.849 | -122.522 | 192 | 2.5 |
| T | Knobcone Point | Contra Costa | 37.842 | -121.896 | 534 | 22.0 |
| T | Las Trampas | Alameda | 37.828 | -122.052 | 507 | 16.0 |
| M | San Bruno Mt | San Mateo | 37.684 | -122.428 | 270 | 3.4 |
| M | Montara Mnt | San Mateo | 37.569 | -122.489 | 347 | 2.3 |
| T | Pulgas Ridge | San Mateo | 37.479 | -122.294 | 193 | 5.3 |
| T | Edgewood | San Mateo | 37.462 | -122.277 | 239 | 5.6 |
| I | Mines Road | Contra Costa | 37.448 | -121.518 | 887 | 37.6 |
| T | Huddart Park | San Mateo | 37.428 | -122.308 | 417 | 10.3 |
| I | Mt Hamilton | Santa Clara | 37.344 | -121.632 | 583 | 30.2 |
| I | San Felipe Ranch | Santa Clara | 37.272 | -121.566 | 1142 | 39.1 |
| T | Butano Ridge | San Mateo | 37.242 | -122.251 | 448 | 12.8 |
| I | Blue Ridge | Santa Clara | 37.230 | -121.538 | 950 | 43.5 |
| T | Castle Rock | Santa Cruz | 37.228 | -122.109 | 850 | 21.0 |
| T | El Sereno | Santa Clara | 37.215 | -122.025 | 756 | 25.6 |
| I | Simon Newman | Merced | 37.211 | -121.187 | 246 | 67.5 |
| T | China Grade | Santa Cruz | 37.198 | -122.198 | 581 | 13.1 |
| T | Sierra Azul | Santa Cruz | 37.193 | -121.958 | 504 | 24.0 |
| T | Upper Gazos | Santa Cruz | 37.189 | -122.272 | 413 | 8.1 |
| T | Mt Sombroso | Santa Clara | 37.178 | -121.905 | 808 | 22.3 |
| I | Willow Ridge | Santa Clara | 37.155 | -121.456 | 687 | 43.8 |
| T | Lockheed Chalks | Santa Cruz | 37.121 | -122.221 | 550 | 5.8 |
| T | Loma Chiquita | Santa Clara | 37.106 | -121.831 | 829 | 16.6 |
| I | Palasou Ridge | Santa Clara | 37.079 | -121.471 | 320 | 38.4 |
| T | Quail Hollow | Santa Cruz | 37.073 | -122.063 | 155 | 12.4 |
| T | Bonny Doon | Santa Cruz | 37.051 | -122.141 | 510 | 6.6 |
| T | Mt Madonna | Santa Cruz | 37.001 | -121.706 | 487 | 15.9 |
| M | Pajaro Hills | Monterey | 36.843 | -121.712 | 149 | 7.7 |
| I | Gabilan Ranch | San Benito | 36.711 | -121.454 | 720 | 31.4 |
| M | Fort Ord NR | Monterey | 36.682 | -121.775 | 38 | 3.4 |
| M | East Fort Ord | Monterey | 36.640 | -121.718 | 66 | 9.6 |
| M | Fort Ord BLM | Monterey | 36.628 | -121.777 | 101 | 5.2 |
| M | Toro Park | Monterey | 36.597 | -121.682 | 181 | 14.4 |
| M | Jack's Peak State Park | Monterey | 36.569 | -121.859 | 178 | 4.1 |
| I | Gloria Road | San Benito | 36.562 | -121.235 | 627 | 53.5 |
| M | Pt Lobos State Reserve | Monterey | 36.509 | -121.929 | 114 | 0.9 |
| I | La Macchia Ranch | Monterey | 36.509 | -121.312 | 639 | 48.7 |
| I | West Pinnacles | San Benito | 36.494 | -121.232 | 585 | 54.2 |
| I | East Pinnacles | San Benito | 36.468 | -121.185 | 563 | 55.5 |
| I | Berry Ranch | San Benito | 36.358 | -120.903 | 995 | 67.0 |
| M | Pt Sur Rock | Monterey | 36.304 | -121.899 | 103 | 0.1 |
| I | Arroyo Seco | Monterey | 36.231 | -121.496 | 294 | 17.3 |
| I | Indians | Monterey | 36.121 | -121.466 | 679 | 12.9 |
| I | Upper Pine Canyon | Monterey | 36.114 | -121.240 | 640 | 26.8 |
| T | Highland Ridge | Monterey | 36.061 | -121.568 | 628 | 2.1 |
| T | Naciemento Rd | Monterey | 36.004 | -121.388 | 499 | 9.6 |
| T | Coast Ridge Rd | Monterey | 35.947 | -121.401 | 912 | 6.5 |
| T | Plaskett Gate | Monterey | 35.932 | -121.432 | 919 | 3.4 |
| M | Pacific Valley | Monterey | 35.920 | -121.462 | 260 | 0.6 |
| T | Lion's Den | Monterey | 35.859 | -121.345 | 945 | 5.4 |
| I | Lynch Canyon | San Luis Obispo | 35.782 | -120.997 | 356 | 22.3 |
| M | Arroyo de la Cruz | San Luis Obispo | 35.728 | -121.306 | 108 | 0.9 |
| T | Cerro Alto | San Luis Obispo | 35.421 | -120.730 | 490 | 12.7 |
| I | Black Mountain | San Luis Obispo | 35.378 | -120.341 | 890 | 38.0 |
| T | Pennington Canyon | San Luis Obispo | 35.364 | -120.708 | 413 | 11.2 |
| M | Hollister Peak | San Luis Obispo | 35.346 | -120.803 | 192 | 2.4 |
| I | Pozo Summit | San Luis Obispo | 35.344 | -120.295 | 824 | 38.9 |
| M | Elfin Forest | San Luis Obispo | 35.334 | -120.827 | 19 | 0.2 |
| T | Cuesta Ridge | San Luis Obispo | 35.321 | -120.605 | 727 | 18.7 |
| I | Rinconada | San Luis Obispo | 35.287 | -120.479 | 598 | 22.0 |
| I | Big Falls Trailhead | San Luis Obispo | 35.278 | -120.490 | 647 | 20.6 |
| I | Hi Mountain Camp | San Luis Obispo | 35.264 | -120.415 | 705 | 25.0 |
| M | Montana de Oro | San Luis Obispo | 35.259 | -120.881 | 110 | 1.2 |
| T | Upper Lopez Canyon | San Luis Obispo | 35.209 | -120.452 | 266 | 19.1 |
| M | Indian Knob | San Luis Obispo | 35.199 | -120.666 | 238 | 4.3 |
| I | Sierra Madre | San Luis Obispo | 35.071 | -120.055 | 875 | 52.3 |
| I | La Brea Canyon | Santa Barbara | 34.945 | -120.106 | 467 | 47.1 |
| M | Point Sal | Santa Barbara | 34.913 | -120.628 | 321 | 2.1 |
| I | Figueroa Mountain | Santa Barbara | 34.741 | -119.996 | 1087 | 31.0 |
| M | Harris Grade | Santa Barbara | 34.729 | -120.435 | 141 | 15.3 |
| M | Burton Mesa ER | Santa Barbara | 34.714 | -120.477 | 118 | 11.4 |
| M | Vandenberg AFB | Santa Barbara | 34.695 | -120.544 | 109 | 5.3 |
| M | Point Arguello | Santa Barbara | 34.614 | -120.557 | 332 | 6.7 |

**Table S2** Total plot occurrence (frequency) and average cover (%) of species and life forms in each climate zone. Names of taxa have been updated to 2nd Edition of the Jepson Manual (Baldwin *et al.* 2012). LF = life form, (T = tree, S = shrub, SS = subshrub, V = vine, PF = perennial forb, PG = perennial grass, F = fern); M = maritime, T = transition, I = interior; with F = frequency or C = cover (average percent).

| **Species** | **LH** | **MF** | **MC** | **TF** | **TC** | **IF** | **IC** |
| --- | --- | --- | --- | --- | --- | --- | --- |
| *Achillea millefolium* L. | PF | 1 | 0.6 | 1 | 0.6 | 2 | 0.5 |
| *Acmispon glaber* (Vogel) Brouillet | PF | 8 | 0.76 | 9 | 2.71 | 13 | 2.76 |
| *Acourtia microcephala* DC. | PF |  |  | 1 | 0.6 | 2 | 0.25 |
| *Adenocaulon bicolor* Hook. | PF |  |  | 1 | 0.1 |  |  |
| *Adenostoma fasciculatum* Hook. & Arn. | S | 16 | 18.61 | 22 | 27.6 | 29 | 47.56 |
| *Adenostoma sparsifolium* Torr. | S |  |  |  |  | 1 | 12.6 |
| *Adiantum jordanii* Müll. Hal. | F |  |  |  |  | 1 | 0.4 |
| *Aesculus californica* (Spach) Nutt. | T |  |  |  |  | 1 | 3.8 |
| *Agrostis hooveri* Swallen | PG | 1 | 2.8 |  |  |  |  |
| *Agrostis pallens* Trin. (*A. diegoensis* Vasey) | PG | 1 | 0.1 |  |  | 1 | 4.5 |
| *Agrostis pallens* Trin. | PG | 3 | 6.67 |  |  |  |  |
| *Agrostis stolonifera* L. | PG | 5 | 2.4 |  |  |  |  |
| *Anaphalis margaritacea* (L.) Benth. | PF | 3 | 0.77 |  |  |  |  |
| *Anthriscus caucalis* M. Bieb. | PF |  |  |  |  | 1 | 2 |
| *Arbutus menziesii* Pursh | T | 3 | 0.97 | 6 | 1.97 |  |  |
| *Arctostaphylos andersonii* A. Gray | S |  |  | 3 | 15.63 |  |  |
| *Arctostaphylos auriculata* Eastw. | S |  |  | 2 | 40.45 |  |  |
| *Arctostaphylos bakeri* Eastw. | S |  |  | 1 | 64.5 |  |  |
| *Arctostaphylos bakeri* Eastw. *subsp. sublaevis* P.V. Wells | S |  |  | 1 | 15.9 |  |  |
| *Arctostaphylos canescens* Eastw. | S |  |  | 2 | 54.25 | 1 | 26 |
| *Arctostaphylos crustacea* Eastw. | S | 8 | 37.19 | 5 | 27.74 | 2 | 45.5 |
| *Arctostaphylos crustacea subsp. crinita* (J.E. Adams) V.T. Parker et al. | S |  |  | 7 | 29.29 |  |  |
| *Arctostaphylos cruzensis* Roof | S | 1 | 55.2 |  |  |  |  |
| *Arctostaphylos edmundsii* J.T. Howell | S | 1 | 41.2 |  |  |  |  |
| *Arctostaphylos gabilanensis* V.T. Parker & M.C. Vasey | S |  |  |  |  | 2 | 12.65 |
| *Arctostaphylos glandulosa Eastw.* | S | 1 | 29.5 | 4 | 16.58 | 3 | 25.27 |
| *Arctostaphylos glandulosa subsp. cushingiana* (Eastw.) J.E. Keeley et al. | S | 1 | 48.1 | 2 | 6.35 | 4 | 28 |
| *Arctostaphylos glandulosa subsp. howellii* (Eastw.) P.V. Wells | S |  |  | 2 | 16.25 | 2 | 13.6 |
| *Arctostaphylos glauca* Lindl. | S | 1 | 12.2 | 1 | 0.5 | 16 | 20.79 |
| *Arctostaphylos glutinosa* B. Schreib. | S |  |  | 1 | 6 |  |  |
| *Arctostaphylos hookeri G. Don* | S | 4 | 20.4 |  |  |  |  |
| *Arctostaphylos hookeri subsp. hearstiorum* (Hoover & Roof) P.V. Wells | S | 1 | 2 |  |  |  |  |
| *Arctostaphylos hooveri* P.V. Wells | S |  |  | 3 | 28.2 |  |  |
| *Arctostaphylos imbricata* Eastw. | S | 1 | 52.9 |  |  |  |  |
| *Arctostaphylos luciana* P.V. Wells | S |  |  | 1 | 31.3 |  |  |
| *Arctostaphylos manzanita* Parry | S |  |  | 1 | 16.2 | 3 | 6.37 |
| *Arctostaphylos manzanita subsp. laevigata* (Eastw.) Munz | S |  |  | 1 | 52.4 |  |  |
| *Arctostaphylos montana* Eastw. | S |  |  | 1 | 58.1 |  |  |
| *Arctostaphylos montaraensis* Roof | S | 1 | 57.6 |  |  |  |  |
| *Arctostaphylos montereyensis* Hoover | S | 3 | 34.53 |  |  |  |  |
| *Arctostaphylos morroensis* Wiesl. & B. Schreib. | S | 1 | 23.2 |  |  |  |  |
| *Arctostaphylos obispoensis* Eastw. | S |  |  | 2 | 29.6 |  |  |
| *Arctostaphylos osoensis* P.V. Wells | S | 1 | 73.7 |  |  |  |  |
| *Arctostaphylos pajaroensis* (J.E. Adams) J.E. Adams | S | 1 | 55 |  |  |  |  |
| *Arctostaphylos pallida* Eastw. | S | 1 | 62.7 |  |  |  |  |
| *Arctostaphylos pechoensis* (Abrams) Eastw. | S | 1 | 34.9 | 1 | 0.3 |  |  |
| *Arctostaphylos pilosula* Jeps. & Wiesl. | S | 1 | 53.5 | 1 | 31 | 1 | 12.8 |
| *Arctostaphylos pumila* Nutt. | S | 2 | 20.85 |  |  |  |  |
| *Arctostaphylos pungens* Kunth | S |  |  |  |  | 1 | 3.3 |
| *Arctostaphylos purissima* P.V. Wells | S | 5 | 43.82 |  |  |  |  |
| *Arctostaphylos rudis* Jeps. & Wiesl. | S | 3 | 18.93 |  |  |  |  |
| *Arctostaphylos sensitiva* Jeps. | S | 1 | 55.5 | 5 | 26.64 |  |  |
| *Arctostaphylos silvicola* Jeps. & Wiesl. | S |  |  | 2 | 48 |  |  |
| *Arctostaphylos stanfordiana* Parry | S |  |  |  |  | 1 | 31.3 |
| *Arctostaphylos tomentosa* (Pursh) Lindl. | S | 6 | 41 |  |  |  |  |
| *Arctostaphylos virgata Eastw.* | S | 1 | 0.1 | 1 | 23.9 |  |  |
| *Arctostaphylos viscida subsp. pulchella* (Howell) P.V. Wells | S |  |  | 1 | 7.6 | 1 | 13.3 |
| *Arctostaphylos x campbelliae* Eastw. (Pro. Sp.) | S |  |  |  |  | 2 | 25.35 |
| *Armeria maritima* (Mill.) Willd. | PF | 1 | 0.4 |  |  |  |  |
| *Artemisia californica* Less. | SS | 9 | 1.44 | 1 | 0.6 |  |  |
| *Artemisia pycnocephala* (Less.) DC. | SS | 1 | 6.2 |  |  |  |  |
| *Aspidotis densa* (Brack.) Lellinger | F |  |  | 1 | 0.5 |  |  |
| *Baccharis pilularis* DC. | S | 15 | 3.71 | 8 | 3.58 | 2 | 0.95 |
| *Bromus carinatus* Hook. & Arn. | PF | 1 | 0.1 |  |  |  |  |
| *Calamagrostis ophitidis* (J.T. Howell) Nygren | S |  |  | 1 | 0.8 |  |  |
| *Calamagrostis rubescens* Buckley | PF |  |  | 2 | 1.4 |  |  |
| *Calochortus albus* (Benth.) Benth. | PF |  |  |  |  | 1 | 0.1 |
| *Calochortus raichei* Farwig & V. Girard | PF |  |  | 1 | 2 |  |  |
| *Calochortus weedii* Alph. Wood | PF |  |  | 1 | 0.2 |  |  |
| *Calystegia collina* (Greene) Brummitt | PF |  |  | 1 | 0.2 |  |  |
| *Calystegia longipes* (S. Watson) Brummitt | PF |  |  |  |  | 1 | 1.2 |
| *Calystegia occidentalis* (A. Gray) Brummitt | PF |  |  | 1 | 0.6 |  |  |
| *Carex brevicaulis* Mack. | PG | 1 | 1 |  |  |  |  |
| *Carex globosa* Boott | PG | 12 | 0.79 | 5 | 1.26 | 1 | 6.8 |
| *Carpobrotus chilensis* (Molina) N.E. Br. | PF | 1 | 2.7 |  |  |  |  |
| *Castilleja foliolosa* Hook. & Arn. | PF | 2 | 0.15 | 1 | 0.1 | 4 | 0.55 |
| *Castilleja latifolia* Hook. & Arn. | PF | 1 | 0.4 |  |  |  |  |
| *Ceanothus cuneatus* Nutt. | S | 4 | 3.98 | 14 | 9.73 | 19 | 15.06 |
| *Ceanothus cuneatus var. fascicularis* (McMinn) Hoover | S | 3 | 11.1 |  |  |  |  |
| *Ceanothus foliosus* Parry | S | 1 | 1 | 1 | 0.8 |  |  |
| *Ceanothus integerrimus* Hook. & Arn. | S |  |  | 1 | 1 |  |  |
| *Ceanothus jepsonii* Greene | S |  |  | 2 | 9.35 |  |  |
| *Ceanothus leucodermis* Greene | S |  |  | 2 | 4.55 | 2 | 0.95 |
| *Ceanothus masonii* McMinn | S |  |  | 1 | 1.2 |  |  |
| *Ceanothus oliganthus var. sorediatus* (Hook. & Arn.) Hoover | S | 2 | 4 | 5 | 2.92 | 6 | 3.98 |
| *Ceanothus papillosus* Torr. & A. Gray | S | 1 | 11.8 | 7 | 8.67 | 2 | 12.95 |
| *Ceanothus prostratus* Benth. | S | 1 | 1.6 |  |  |  |  |
| *Ceanothus rigidus* Nutt. | S | 2 | 3.15 |  |  |  |  |
| *Ceanothus sonomensis* J.T. Howell | S |  |  |  |  | 1 | 14.1 |
| *Ceanothus thyrsiflorus* Eschsch. | S | 6 | 9.4 |  |  |  |  |
| *Ceanothus thyrsiflorus var. griseus* Trel. | S | 1 | 2.7 |  |  |  |  |
| *Cercocarpus betuloides* Nutt. | S |  |  | 5 | 4.82 | 5 | 5.28 |
| *Chlorogalum pomeridianum* (DC.) Kunth | T | 1 | 0.3 | 4 | 1.6 | 12 | 1.28 |
| *Chrysolepis chrysophylla* (Hook.) Hjelmq. | S | 2 | 36.35 | 3 | 6.1 |  |  |
| *Cirsium occidentale* (Nutt.) Jeps. | V | 1 | 0.2 |  |  |  |  |
| *Clematis lasiantha* Nutt. | PF |  |  |  |  | 9 | 4.89 |
| *Clinopodium douglasii* (Benth.) Kuntze | PF | 4 | 0.5 | 1 | 0.1 |  |  |
| *Conicosia pugioniformis* (L.) N.E. Br. | PF | 1 | 0.2 |  |  |  |  |
| *Corethrogyne filaginifolia* (Hook. & Arn.) Nutt. | PF | 3 | 0.7 |  |  |  |  |
| *Cortaderia jubata* (Lemoine) Stapf | PG | 1 | 0.9 |  |  |  |  |
| *Croton californicus* Müll. Arg. | PF | 1 | 0.9 |  |  |  |  |
| *Delairea odorata* Lem. | PF | 1 | 0.1 |  |  |  |  |
| *Dendromecon rigida* Benth. | S | 3 | 3.47 | 3 | 6.8 | 7 | 4.76 |
| *Dichelostemma capitatum* (Benth.) Alph. Wood | PF | 1 | 0.3 |  |  |  |  |
| *Dichelostemma volubile* (Kellogg) A. Heller | SS |  |  | 1 | 0.5 | 1 | 0.6 |
| *Dryopteris arguta* (Kaulf.) Maxon | F | 7 | 1.24 | 1 | 1.3 |  |  |
| *Dudleya caespitosa* (Haw.) Britton & Rose | PF | 4 | 0.65 |  |  |  |  |
| *Dudleya pulverulenta* (Nutt.) Britton & Rose | PF | 1 | 0.1 |  |  |  |  |
| *Elymus condensatus* J. Presl | PG | 1 | 0.3 |  |  | 3 | 1.07 |
| *Elymus triticoides* Buckley | PG | 1 | 0.6 |  |  |  |  |
| *Ericameria arborescens* (A. Gray) Greene | SS |  |  | 2 | 0.95 | 1 | 3 |
| *Ericameria ericoides* (Less.) Jeps. | SS | 6 | 8.02 | 1 | 14.5 |  |  |
| *Ericameria linearifolia* (DC.) Urbatsch & Wussow | SS |  |  | 2 | 3.2 | 2 | 2.1 |
| *Erigeron foliosus* Nutt. | PF |  |  |  |  | 1 | 0.1 |
| *Erigeron glaucus* Ker Gawl. | PF | 1 | 7.3 |  |  |  |  |
| *Eriodictyon californicum* (Hook. & Arn.) Torr. | SS | 2 | 1.5 | 8 | 2.04 | 5 | 3.04 |
| *Eriodictyon tomentosum* Benth. | SS |  |  |  |  | 3 | 6.13 |
| *Eriogonum fasciculatum* Benth. | SS |  |  |  |  | 5 | 3.92 |
| *Eriogonum gracile* Benth. | PF |  |  |  |  | 1 | 0.1 |
| *Eriogonum latifolium* Sm. | PF | 1 | 1 |  |  |  |  |
| *Eriogonum parvifolium* Sm. | PF | 2 | 0.65 |  |  |  |  |
| *Eriophyllum confertiflorum* (DC.) A. Gray | SS | 6 | 0.7 | 7 | 1.09 | 6 | 0.85 |
| *Eriophyllum staechadifolium* Lag. | SS | 2 | 11.3 |  |  |  |  |
| *Erysimum capitatum* (Hook.) Greene | PF |  |  | 1 | 0.2 |  |  |
| *Eschscholzia californica* Cham. | PF |  |  | 2 | 0.35 |  |  |
| *Eurybia radulina* (A. Gray) G.L. Nesom | PF |  |  |  |  | 3 | 2.13 |
| *Fragaria chiloensis* (L.) Mill. | PF | 1 | 0.1 |  |  |  |  |
| *Frangula californica* (Eschsch.) A. Gray | S | 7 | 6.37 | 10 | 5.48 | 2 | 2.55 |
| *Fraxinus dipetala* Hook. & Arn. | S |  |  |  |  | 1 | 1.3 |
| *Galium andrewsii* A. Gray | PF | 1 | 0.3 | 1 | 0.9 | 4 | 2.38 |
| *Galium californicum* Hook. & Arn. | PF | 4 | 0.2 | 4 | 0.43 | 10 | 1.09 |
| *Galium hardhamiae* Dempster | PF |  |  | 1 | 1 |  |  |
| *Galium porrigens* Dempster | PF | 2 | 1.25 | 7 | 1.07 | 9 | 0.49 |
| *Garrya elliptica* Lindl. | S | 2 | 0.6 | 3 | 2 | 2 | 5.05 |
| *Garrya fremontii* Torr. | S |  |  |  |  | 3 | 15.77 |
| *Garrya veatchii* Kellogg | S |  |  |  |  | 1 | 0.3 |
| *Genista monspessulana* (L.) L.A.S. Johnson | S | 2 | 4.35 |  |  |  |  |
| *Hazardia squarrosa* (Hook. & Arn.) Greene | SS | 1 | 0.3 |  |  | 1 | 1.3 |
| *Helianthella californica* A. Gray | PF |  |  |  |  | 2 | 3.25 |
| *Helianthemum scoparium* Nutt. | SS | 7 | 0.94 | 3 | 3.7 | 5 | 1.12 |
| *Heracleum maximum* W. Bartram | PF | 1 | 0.3 |  |  |  |  |
| *Hesperocyparis abramsiana* (C.B. Wolf) Bartel | T |  |  | 2 | 40.25 |  |  |
| *Hesperocyparis goveniana* (Gordon) Bartel | T | 1 | 60.7 |  |  |  |  |
| *Hesperocyparis sargentii* (Jeps.) Bartel | T |  |  | 4 | 30.95 |  |  |
| *Hesperoyucca whipplei* (Torr.) Trel. | PF |  |  | 1 | 8.6 | 2 | 0.75 |
| *Heteromeles arbutifolia* (Lindl.) M. Roem. | S | 16 | 3.26 | 20 | 3.46 | 13 | 12.62 |
| *Horkelia cuneata* Lindl. | PF | 5 | 0.72 |  |  |  |  |
| *Horkelia cuneata var. puberula* (Rydb.) Ertter & Reveal | PF | 1 | 3.3 | 1 | 4.4 | 1 | 1.1 |
| *Horkelia cuneata var. sericea* (A. Gray) Ertter & Reveal | PF | 3 | 0.73 |  |  |  |  |
| *Hypericum concinnum* Benth. | PF |  |  |  |  | 2 | 3.45 |
| *Iris douglasiana* Herb. | PF | 2 | 0.7 |  |  |  |  |
| *Iris fernaldii* R.C. Foster | PF |  |  |  |  | 1 | 0.1 |
| *Keckiella breviflora* (Lindl.) Straw | SS |  |  | 1 | 0.1 | 2 | 4.4 |
| *Kopsiopsis strobilacea* (A. Gray) Beck | PF |  |  |  |  | 1 | 0.1 |
| *Lepechinia calycina* (Benth.) Munz | SS | 3 | 1.43 | 5 | 1.98 | 6 | 2.2 |
| *Lobularia maritima* (L.) Desv. | PF | 1 | 0.4 |  |  |  |  |
| *Lomatium dasycarpum* (Torr. & A. Gray) J.M. Coult. & Rose | PF |  |  | 1 | 1 | 2 | 0.2 |
| *Lonicera hispidula* (Lindl.) Torr. & A. Gray | V | 2 | 0.15 | 2 | 3.3 | 2 | 1.05 |
| *Lonicera interrupta* Benth. | S |  |  |  |  | 4 | 1.2 |
| *Lonicera subspicata* Hook. & Arn. | S |  |  | 1 | 1.2 | 3 | 6.8 |
| *Maianthemum racemosum* (L.) Link | PF | 1 | 0.1 |  |  |  |  |
| *Marah fabacea* (Naudin) Greene | V | 3 | 2.23 | 4 | 1.25 | 8 | 1.94 |
| *Melica imperfecta* Trin. | PG | 1 | 1 | 2 | 0.9 | 2 | 1.95 |
| *Melica torreyana* Scribn. | PG |  |  | 1 | 2 | 3 | 3.17 |
| *Mimulus aurantiacus* Curtis | SS | 19 | 6.09 | 16 | 5.38 | 10 | 4.75 |
| *Monardella villosa* Benth. | PF |  |  | 2 | 0.4 | 3 | 2.1 |
| *Notholithocarpus densiflorus* (Hook. & Arn.) Manos et al. | T |  |  | 3 | 2.17 |  |  |
| *Oxalis pes-caprae* L. | PF | 1 | 0.1 |  |  |  |  |
| *Paeonia californica* Nutt. | PF | 2 | 1.5 | 1 | 0.3 | 2 | 2.25 |
| *Pedicularis densiflora* Hook. | PF | 1 | 0.1 | 2 | 3.1 | 6 | 4.27 |
| *Pellaea andromedifolia* (Kaulf.) Fée | F |  |  | 1 | 0.1 | 2 | 0.5 |
| *Pellaea mucronata* (D.C. Eaton) D.C. Eaton | F | 1 | 0.4 | 1 | 0.4 | 3 | 1.57 |
| *Pentagramma triangularis* (Kaulf.) Yatsk., Windham, & E. Wollenw. | F | 3 | 0.6 | 1 | 1.8 | 9 | 2.17 |
| *Phoradendron bolleanum* (Seem.) Eichler | PF |  |  | 3 | 0.77 |  |  |
| *Phoradendron leucarpum* (Raf.) Reveal & M.C. Johnst | PF |  |  |  |  | 2 | 0.2 |
| *Pickeringia montana* Nutt. | S | 1 | 0.3 | 7 | 2.93 | 4 | 6.25 |
| *Pinus attenuata* Lemmon | T |  |  | 9 | 5.93 |  |  |
| *Pinus coulteri* D. Don | T |  |  | 3 | 10.8 | 2 | 1.9 |
| *Pinus muricata* D. Don | T | 3 | 14.33 |  |  |  |  |
| *Pinus ponderosa* Lawson & C. Lawson | T |  |  | 2 | 3.5 |  |  |
| *Pinus radiata* D. Don | T | 1 | 5.8 | 1 | 1.3 |  |  |
| *Pinus sabiniana* D. Don | T |  |  |  |  | 8 | 3.4 |
| *Piperia elegans* (Lindl.) Rydb. | PF | 3 | 0.43 | 2 | 0.1 |  |  |
| *Piperia leptopetala* Rydb. | PF |  |  | 1 | 0.6 |  |  |
| *Poa secunda* J. Presl | PG | 1 | 0.6 |  |  |  |  |
| *Polygala californica* Nutt. | PF | 2 | 0.8 | 3 | 0.5 |  |  |
| *Polystichum munitum* (Kaulf.) C. Presl | PG | 2 | 0.1 | 1 | 1.3 |  |  |
| *Prunus ilicifolia* (Hook. & Arn.) D. Dietr. | S | 2 | 0.95 | 2 | 3.7 | 4 | 5.25 |
| *Pseudognaphalium beneolens* (Davidson) Anderb. | PF |  |  | 1 | 0.1 |  |  |
| *Pseudognaphalium californicum* (DC.) Anderb. | PF | 2 | 0.35 | 1 | 0.1 |  |  |
| *Pseudognaphalium microcephalum* (Nutt.) Anderb. | PF | 1 | 0.3 |  |  |  |  |
| *Pseudognaphalium ramosissimum* (Nutt.) Anderb. | PF | 1 | 0.1 |  |  |  |  |
| *Pseudotsuga menziesii* (Mirb.) Franco | T | 1 | 0.6 | 5 | 3.98 |  |  |
| *Pteridium aquilinum* (L.) Kuhn | F | 7 | 8.24 | 4 | 11.88 |  |  |
| *Quercus agrifolia* Née | T | 10 | 7.52 | 6 | 3.57 | 2 | 5.7 |
| *Quercus berberidifolia* Liebm. | T |  |  | 3 | 8.93 | 11 | 20.38 |
| *Quercus chrysolepis* Liebm. | T | 1 | 9.6 | 5 | 7.24 | 1 | 7.8 |
| *Quercus douglasii* Hook. & Arn. | T |  |  |  |  | 2 | 7.45 |
| *Quercus durata* Jeps. | S |  |  | 5 | 21.9 | 2 | 20.25 |
| *Quercus john-tuckeri* Nixon & C.H. Mull. | T |  |  | 1 | 0.1 |  |  |
| *Quercus kelloggii* Newb. | T |  |  | 2 | 1.8 | 1 | 0.3 |
| *Quercus wislizeni* DC. | T | 8 | 13.99 | 16 | 6.03 | 8 | 10.1 |
| *Rhamnus crocea* Nutt. | S | 3 | 1.77 | 4 | 4.4 | 7 | 3.57 |
| *Ribes malvaceum* Sm. | S |  |  |  |  | 1 | 0.1 |
| *Ribes menziesii* Pursh | S | 1 | 0.3 | 1 | 0.1 | 1 | 0.1 |
| *Ribes sanguineum var. glutinosum* (Benth.) Loudon | S | 1 | 0.1 |  |  |  |  |
| *Ribes speciosum* Pursh | S | 2 | 2.15 |  |  |  |  |
| *Rosa gymnocarpa* Nutt. | S |  |  | 2 | 1.15 | 1 | 1.9 |
| *Rubus ursinus* Cham. & Schltdl. | V | 5 | 3.3 | 1 | 4.3 |  |  |
| *Salvia mellifera* Greene | SS | 11 | 6.26 | 7 | 11.19 | 5 | 3.42 |
| *Salvia sonomensis* Greene | SS |  |  | 3 | 10.6 | 3 | 11.73 |
| *Salvia spathacea* Greene | PF | 3 | 2.07 | 2 | 0.75 | 3 | 9 |
| *Sambucus nigra* L. *subsp. caerulea* (Raf.) Bolli | S |  |  | 1 | 10.6 | 2 | 1 |
| *Sanicula crassicaulis* DC. | PF | 1 | 5.7 | 2 | 0.4 | 3 | 0.77 |
| *Sanicula laciniata* Hook. & Arn. | PF | 1 | 0.3 |  |  |  |  |
| *Scrophularia atrata* Pennell | PF | 1 | 0.2 |  |  |  |  |
| *Scrophularia californica* Cham. & Schltdl. | PF | 1 | 0.3 |  |  |  |  |
| *Scutellaria tuberosa* Benth. | PF |  |  |  |  | 1 | 0.1 |
| *Sequoia sempervirens* (D. Don) Endl. | T | 2 | 1.6 | 3 | 4.97 |  |  |
| *Silene laciniata subsp. californica* (Durand) J.K. Morton | PF | 1 | 1.1 |  |  |  |  |
| *Solanum umbelliferum* Eschsch. | SS |  |  | 1 | 1.2 | 1 | 1.6 |
| *Spergularia macrotheca* (Cham. & Schltdl.) Heynh. | PF | 1 | 0.1 |  |  |  |  |
| *Stachys ajugoides* Benth. | PF | 1 | 0.1 | 1 | 0.1 | 2 | 3 |
| *Stachys bullata* Benth. | PF | 1 | 1.3 |  |  |  |  |
| *Stipa lepida* Hitchc. | PG |  |  | 3 | 3.03 | 7 | 1.73 |
| *Stipa pulchra* Hitchc. | PG | 2 | 1.55 | 1 | 1.1 |  |  |
| *Symphoricarpos mollis* Nutt. | S | 4 | 2.3 | 3 | 0.47 | 2 | 0.35 |
| *Tauschia kelloggii* (A. Gray) J.F. Macbr. | PF |  |  |  |  | 1 | 0.1 |
| *Toxicodendron diversilobum* (Torr. & A. Gray) Greene | S | 14 | 4.55 | 13 | 10.25 | 11 | 9.55 |
| *Toxicoscordion fremontii* (Torr.) Rydb. | PF | 3 | 0.87 | 4 | 1.95 | 3 | 0.77 |
| *Trichostema lanatum* Benth. | PF |  |  | 2 | 1.45 | 4 | 3.9 |
| *Trientalis latifolia* Hook. | PF |  |  | 2 | 2.35 |  |  |
| *Triteleia laxa* Benth. | PF |  |  |  |  | 2 | 0.15 |
| *Umbellularia californica* (Hook. & Arn.) Nutt. | T | 1 | 0.1 | 2 | 1.15 | 3 | 8.93 |
| *Vaccinium ovatum* Pursh | S | 8 | 18.45 | 4 | 30.43 |  |  |
| *Vicia gigantea* Hook. | PF | 1 | 0.1 |  |  |  |  |
| *Wyethia angustifolia* (DC.) Nutt. | PF |  |  |  |  | 1 | 0.2 |
| *Xerophyllum tenax* (Pursh) Nutt. | PF | 1 | 24.5 |  |  |  |  |

**Table S3** Environmental variables sorted by plot, soil, and climate classes. PC1, PC2, and PC3 are the three principle components. Eigenvalue loadings > 0.500 or < 0.500 are bolded. Abbr = abbreviations used in the text.

| **Class** | **Variable** | **Abbr** | **PC1** | **PC2** | **PC3** |
| --- | --- | --- | --- | --- | --- |
| Plot | Elevation (m) | *Elev* | **-0.744** | -0.136 | 0.056 |
| Plot | Distance from coast (km) | *Dist* | **-0.864** | 0.168 | -0.161 |
| Plot | Latitude (o ) | *Lat* | -0.079 | **-0.710** | -0.096 |
| Plot | Longitude (o ) | *Long* | -0.242 | **0.730** | 0.037 |
| Plot | Slope (%) | *Slope* | -0.189 | -0.028 | 0.300 |
| Plot | Aspect (%) | *Aspect* | -0.045 | 0.300 | -0.400 |
| Soil | Clay (%) | *Clay* | -0.193 | 0.103 | **0.701** |
| Soil | Silt (%) | *Silt* | -0.223 | -0.349 | **0.609** |
| Soil | Sand (%) | *Sand* | 0.106 | 0.050 | **-0.643** |
| Soil | Organic matter (%) | *OM* | 0.169 | -0.053 | -0.363 |
| Soil | Nitrogen (mg kg-1 ) | *N* | -0.244 | -0.371 | -0.032 |
| Soil | Phosphorus (mg kg-1 ) | *P* | -0.155 | **-0.593** | -0.155 |
| Soil | Sulfur (mg kg-1) | *S* | 0.275 | **0.635** | 0.131 |
| Soil | PH | *PH* | -0.167 | -0.183 | **-0.655** |
| Soil | Total exchange capacity (meq 100 g-1 ) | *TEC* | -0.278 | -0.253 | 0.353 |
| Soil | Sodium (mg kg-1 ) | *Na* | 0.073 | **-0.565** | 0.177 |
| Soil | Potasium (mg kg-1 ) | *K* | **-0.666** | 0.081 | 0.203 |
| Soil | Calcium (mg kg-1 ) | *Ca* | 0.180 | **-0.633** | 0.207 |
| Soil | Magnesium (mg kg-1 ) | *Mg* | -0.286 | -0.452 | 0.273 |
| Soil | Iron (mg kg-1) | *Fe* | -0.531 | -0.019 | -0.224 |
| Soil | Aluminum (mg kg-1) | *Al* | -0.188 | 0.227 | **-0.506** |
| Climate | Atmospheric water potential (MPa) | *Ψ* atm | **0.896** | 0.041 | 0.016 |
| Climate | Vapor pressure deficit (kPa) | *VPD* | **-0.923** | -0.112 | -0.030 |
| Climate | Cloud frequency (%) | *CF* | **0.789** | 0.150 | 0.046 |
| Climate | Potential evapotranspiration (mm day-1 ) | *PET* | **-0.905** | -0.026 | -0.057 |
| Climate | Maximum dry season average temperature (^o^ C) | *T_max_* | **-0.857** | -0.014 | -0.072 |
| Climate | Mean temperature warmest month ^(o^ C) | *MTW* | **-0.910** | 0.046 | -0.117 |
| Climate | Temperature seasonality (^o^ C) | *TS* | **-0.905** | -0.169 | -0.114 |
| Climate | Minimum wet season average temperature (^o^ C) | *T_min_* | **0.525** | -0.417 | -0.107 |
| Climate | Mean temperature coldest month (^o^ C) | *MTC* | **0.870** | -0.128 | -0.015 |
| Climate | Precipitation (mm) | *Precip* | -0.06 | **-0.665** | 0.170 |

**Fig. S1.** Group average cluster analysis of 87 plots based on 10 climate variables with high eigenvalues from the PCA. Climate variables were normalized and Euclidiean distances used to calculate a resemblance distance matrix. Dark circles represent maritime (coastal lowland, n=25), light gray triangles represent transition (coastal upland, n=32), and white squares represent interior (n=30) climate zone groups.

**Maritime (25)**

**Transition (32)**

**Interior (30)**
